# Notching on Cancer’s Door: Notch Signaling in Brain Tumors

**DOI:** 10.3389/fonc.2014.00341

**Published:** 2015-01-05

**Authors:** Marcin Teodorczyk, Mirko H. H. Schmidt

**Affiliations:** ^1^Molecular Signal Transduction Laboratories, Institute for Microscopic Anatomy and Neurobiology, Focus Program Translational Neuroscience (FTN), Rhine Main Neuroscience Network (rmn^2^), Johannes Gutenberg University of Mainz School of Medicine, Mainz, Germany

**Keywords:** brain tumor therapy, clinical trials, glioma, medulloblastoma, Notch signaling, stem-like brain tumor-propagating cells

## Abstract

Notch receptors play an essential role in the regulation of central cellular processes during embryonic and postnatal development. The mammalian genome encodes for four Notch paralogs (Notch 1–4), which are activated by three Delta-like (Dll1/3/4) and two Serrate-like (Jagged1/2) ligands. Further, non-canonical Notch ligands such as epidermal growth factor like protein 7 (EGFL7) have been identified and serve mostly as antagonists of Notch signaling. The Notch pathway prevents neuronal differentiation in the central nervous system by driving neural stem cell maintenance and commitment of neural progenitor cells into the glial lineage. Notch is therefore often implicated in the development of brain tumors, as tumor cells share various characteristics with neural stem and progenitor cells. Notch receptors are overexpressed in gliomas and their oncogenicity has been confirmed by gain- and loss-of-function studies *in vitro* and *in vivo*. To this end, special attention is paid to the impact of Notch signaling on stem-like brain tumor-propagating cells as these cells contribute to growth, survival, invasion, and recurrence of brain tumors. Based on the outcome of ongoing studies *in vivo*, Notch-directed therapies such as γ-secretase inhibitors and blocking antibodies have entered and completed various clinical trials. This review summarizes the current knowledge on Notch signaling in brain tumor formation and therapy.

## Introduction

Malignant gliomas represent the most futile type of brain tumor in adults with an annual incidence of 5 per 100,000 individuals (1, 2). According to the World Health Organization (WHO) guidelines, they can be characterized as astrocytomas, oligodendrogliomas, ependymomas, or oligo-astrocytomas (mixed gliomas). Another WHO classification is based on the malignancy of the neoplasms and ranges from grade I, corresponding to low-proliferative non-invasive tumors, up to grade IV, assigned to cytologically malignant, highly infiltrative, mitotically active, and necrosis-prone glioblastoma multiforme (GBM, malignant glioma). Most of the diagnosed GBMs (90–95%) are primary tumors, although secondary glioblastomas might also arise from low-grade tumors (3, 4). Medulloblastomas, the most frequent neoplasms in children, are of a cerebellar origin and are therefore not included in this glioma classification system (5). WHO grades reflect patients’ prognosis: grade I tumors are generally curable by surgical resection alone (1), while the current standard care for GBM involves maximal surgical resection followed by temozolomide (TMZ) chemotherapy and radiation. Unfortunately, this treatment regimen has severe side-effects and barely extends the median survival from 12.1 to 14.6 months (6). Currently, the genetics and molecular biology of brain tumors are the focus of extensive studies. It is well established that glioma-driving mutations affect pathways regulating cellular parameters such as cell growth, apoptosis, migration, and angiogenesis (7–9). Master regulators of these biological processes frequently mutated in glioma are *TP53, PTEN, PDGFR, NF1* or epidermal growth factor receptor (*EGFR*) and there is growing evidence that developmental signaling cues such as Notch are deregulated in malignant brain tumors as well.

## Mammalian Notch Receptors and Ligands

Notch receptors are single-pass transmembrane proteins formed by two non-covalently associated polypeptide chains. The *Drosophila melanogaster* genome encodes only a single Notch gene, but four receptors (Notch 1–4) are found in mammals. After the synthesis of a single-chain precursor, the receptor undergoes a so-called S1 cleavage mediated by furin-like proteases in the trans-Golgi network. S1 generates an N-terminal extracellular domain (NECD) and a C-terminal fragment corresponding to the transmembrane domain (NTM) extending into the cytoplasm (intracellular Notch domain, NICD). The resulting heterodimer, held together by non-covalent bonds, is inserted into the plasma membrane (10). The NECD consists of multiple EGF-like repeats, which partially bind calcium ions and are required for ligand interaction (11). The Notch1 receptor contains 36 EGF repeats in the intracellular domain (12), while Notch2 contains 35 repeats (13), Notch3 34 repeats (14), and Notch4 29 repeats (15). The NECD negative regulatory region (NRR) is composed of three cysteine-rich Lin12/Notch repeats (LNR) (16) and a juxtamembrane heterodimerization domain. As the name suggests, NRR is responsible for the auto-inhibition of the Notch receptor (17, 18) and binds to a short extracellular region of NTM (19). The intracellular domain NICD of the Notch receptor is involved in cellular signaling and includes the recombination signal-binding protein Jκ (RBP-Jκ) associated module (RAM) (20), seven ankyrin (ANK) repeats (21), two nuclear-localization signals (NLS) (22), a transactivation domain (TAD) (23), and a C-terminal PEST sequence (rich in proline, glutamic acid, serine, and threonine) (24).

The canonical Notch ligands belong to the so-called Delta-Serrate-Lag2 (DSL) family and include the five mammalian type I transmembrane proteins Delta-like 1 (Dll1) (25), Dll3 (26), Dll4 (27), Jagged1 (28), and Jagged2 (29). The N-terminal region, the DSL domain and the first two EGF-like repeats are necessary for the interaction with EGF-like repeats of Notch receptors (30, 31). In addition, several transmembrane and soluble proteins have been described as non-canonical ligands, e.g., F3/contactin (32), Delta-like 1 (Dlk1), Dlk2, Delta and Notch-like EGF-related receptor (DNER), or the EGF-like protein 7 (EGFL7) (33–35). Common structural features of this group are the presence of EGF-like repeats and the absence of DSL domain. Dlk1, Dlk2, and DNER are transmembrane proteins (although Dlk1 and Dlk2 also exist in soluble forms), while EGFL7 is a *bona fide* secreted factor. Interestingly, DNER stimulates Notch signaling while current evidence indicates an inhibitory function of Dlk1/2 and EGFL7 (36).

## Notch Signaling Pathway

**Figure 1:**
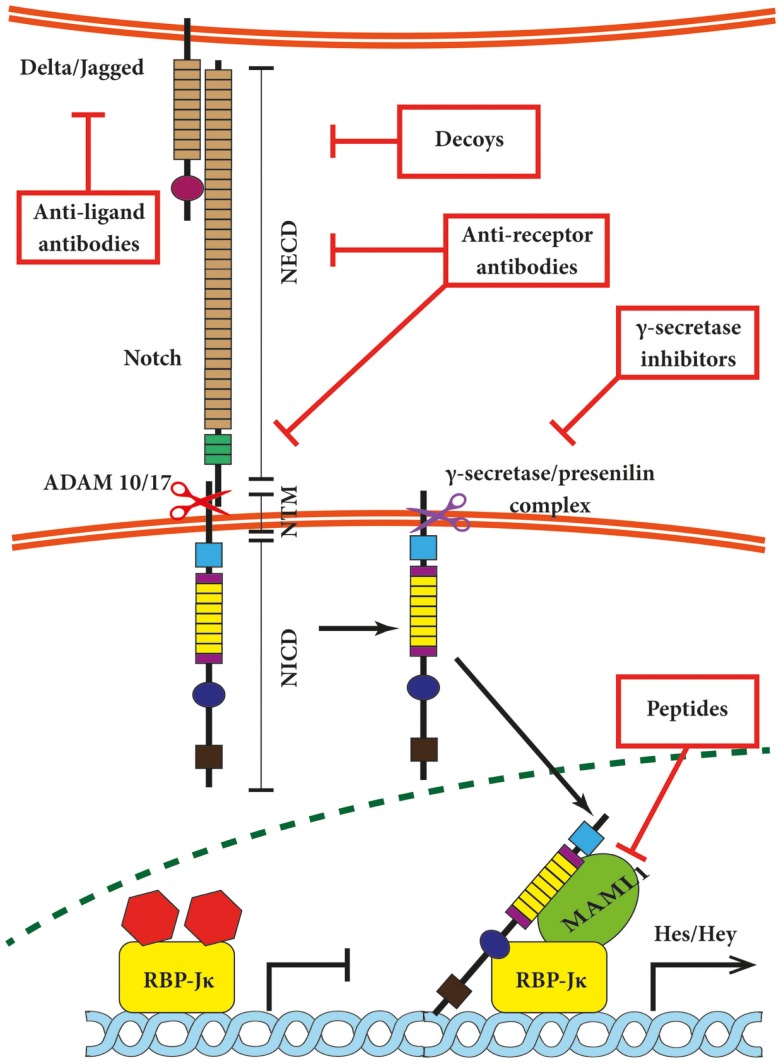
**Canonical Notch signaling with points of intervention of current therapies**. The interaction between Delta/Jagged-type ligands and Notch receptors leads to S2 cleavage on the extracellular site by “a disintegrin and metalloprotease” 10 (ADAM10) or ADAM17, which is followed by S3 cleavage by the γ-secretase–presenilin complex. The S3 cleavage gives rise to an intracellular Notch fragment (NICD) that translocates into the nucleus, where NICD binds to a protein complex containing recombination signal-binding protein Jκ (RBP-Jκ). This mediates the conversion of RBP-Jκ from a repressor to a transcriptional activator and is followed by the recruitment of the co-activator mastermind-like 1 (MAML1). These events lead to the de-repression of transcription of hairy/enhancer of split (Hes) and Hey. Several stages of the Notch signaling pathway are prone to pharmacological intervention and are labeled in the figure. Gamma-secretase inhibitors and blocking antibodies are already in clinical trials and decoys have been tested in animal models. Peptide inhibitors represent potential future treatment modalities. NECD, Notch extracellular domain; NTM, Notch transmembrane domain.

Both Notch receptors and canonical ligands are transmembrane proteins, thus requiring close proximity of the plasma membranes in which they are embedded for interaction. The interaction between neighboring cells is referred to as *in-trans* interaction and switches Notch signaling on (Figure 1). This type of association relies on the EGF-like repeats 11 + 12 of Notch1/2/4 and repeats 10 + 11 of Notch3, respectively (11, 36). *In-cis* interaction between receptors and ligands expressed on the same cell inhibit the Notch pathway (37–39) and involves the EGF-like repeats 24–29 of Notch1 receptor (40). *In-trans* activation triggers the ubiquitination and internalization of the respective ligand and disrupts the hydrophobic interactions between NECD and NTM in the Notch receptor. This in turn exposes NTM to the extracellular S2 cleavage by “a disintegrin and metalloprotease” 10 (ADAM10) or ADAM17 (41). The phenotype of ADAM10 knock-out mice resembles Notch deficiencies (42, 43); however, cell culture-based experiments indicate that ADAM10 and 17 may share substrates including Notch receptors *in vitro* (44, 45). Both proteases create an intermediate membrane-tethered Notch extracellular truncation (NEXT), which is subsequently processed by the γ-secretase–presenilin complex (19). This so-called S3 cleavage releases the intracellular Notch domain NICD, which translocates into the nucleus (46) and binds to a protein complex containing DNA-binding proteins of the CSL family (RBP-Jκ/CBF-1/KBF2 in mammals) and mediates its conversion from a repressor to an activator of transcription followed by the recruitment of the co-activator mastermind-like 1 (MAML1) (47). In turn, the NICD–RBP-Jκ–MAML1 ternary complex recruits further components of the RNA polymerase II holoenzyme such as the histone acetyltransferases CBP/p300 (48) or PCAF/GCN5 (49). Ultimately, these events lead to the transcriptional de-repression of several genes that are often themselves transcriptional repressors such as Hairy/Enhancer of Split (Hes) and Hey (subfamily of Hes, related with YRPW motif) proteins (50–52). Hes-1, Hes-5, and Hey-1 are well-described direct Notch targets (53, 54), and growing evidence suggests Hes-7, Hey-2, and Hey-L as direct target genes (55). The list of genes regulated by Notch is still expanding and includes transcription factors such as NFκB (56, 57), PPAR (58), c-Myc (59–61), Sox2 (62), Pax6 (63), as well as cell cycle regulators such as cyclin D1 (64), and p21/Waf1 (65) among many others.

## Role of Notch Signaling in the Healthy Developing Brain

Notch signaling is an evolutionary conserved pathway that prevents equipotent cells from acquiring identical cell fates. This can be accomplished through the so-called lateral inhibition; a process in which a cell that stochastically acquires enhanced ligand expression stimulates neighboring cells. The *in-cis* inhibition of Notch on the ligand-expressing cells renders this interaction unilateral. In neural development, the signal-sending cell will differentiate into a neuronal precursor while the signal receiving cell will remain as an uncommitted progenitor. This correlates with a decreased expression of Hes-1, Hes-5, and proneural genes as well as Notch ligands in uncommitted progenitors (66, 67).

Notch signaling plays a pivotal role in biological processes including apoptosis, cell proliferation, differentiation, and cell lineage decision in stem cells. Therefore, Notch governs embryonic development and is highly active in undifferentiated cells of the embryonic central nervous system, while its expression is reduced and spatially restricted in the adult brain (66, 68, 69). The relevance of Notch is in part due to its impact on the maintenance of neural stem (NSCs) and progenitor cells (NPCs) as well as the stimulation of their glial differentiation at the expense of their neuronal fate (70–72). Notch1 knock-out mice die before E11.5, approximately the time of neuronal maturation, among other reasons due to a loss of neuroblasts and premature neuronal differentiation (73, 74).

## Role of Notch in Brain Neoplasms

Due to the central role of Notch in differentiation, its deregulation leads to multiple malignancies. The first evidence of the tumorigenic potential of Notch came from the translocation *t*(7; 9) in T cell acute lymphoblastic leukemia (T-ALL), which leads to a fusion of genes encoding the β chain of T cell receptors and the *TAN1/NOTCH1* gene. The product of this gene acts like as a constitutively active form of NICD (75). Subsequently, the components of the Notch pathway have been described to be deregulated in numerous hematological malignancies and brain tumors including gliomas and medulloblastomas (76). The frequency and intensity of Notch2 expression in medulloblastoma is higher than that of Notch1 (77, 78). Moreover, Notch2 was shown to promote tumorigenesis of medulloblastoma, whereas, Notch1 inhibited tumor growth (77). In glioma, however, the correlation between tumor grade and expression of Notch isoforms has not been fully clarified, yet. The tumor-suppressive role of Notch1 is supported by the fact that it has been detected in all gliomas but a subset of grade IV tumors (79). Along the line, Notch1 expression has been shown to be higher in grade II and III malignancies than in glioblastomas (80). Furthermore, nuclear Notch1 staining has been correlated with a better outcome in high-grade glioma subtypes (81). These data suggest a favorable prognosis for patients carrying Notch1-positive tumors. However, according to other reports, the expression of Notch1 exhibits a positive correlation with glioma progression (82, 83), and high expression of Notch1 protein has been reported to be an independent predictor of poor survival in glioma (82). The oncogenic potential of Notch2 is indicated by the fact that the loss of Notch2 positively correlated with a favorable prognosis in small groups of patients diagnosed with oligodendroglioma and GBM (84). However, this conclusion has not yet been fully supported by experimental data.

Various other components of the Notch pathway have been used as markers for different stages of glioma. Transcription of Dll1 has been described to be regulated by the neurogenic transcription factor Hash-1 (85). Both Hash-1 and Dll1 has been found to be upregulated in progressive astrocytoma (grade II and III) as well as in secondary GBM, accompanied by a decrease in the Notch target gene Hes-1. These data indicate that enhanced Dll1 expression inhibits Notch signaling in these subgroups of gliomas. In primary GBM, on the other hand, there is the opposite pattern of Hash-1 and Hes-1 expression (86), implying that enhanced Dll1 expression inhibits Notch signaling while active Notch characterizes primary GBM. Interestingly, Phillips et al. proposed a different classification of gliomas from that of the WHO (81), in which the tumor is assigned into one of three subtypes based on genomic data: proneural, proliferative, or mesenchymal. In terms of prognosis, the proneural group correlates with longer survival as compared to the other two groups. This group contains most of grade III and secondary grade IV tumors (87) and displays the expression of neuroblast and developing neurons markers including Hash-1 and Dll1. These data are in accordance with other publications that show that Notch is active in primary GBM, while low-grade astrocytomas express the ligands Dll1 and Jagged1 (88, 89).

**Figure 2:**
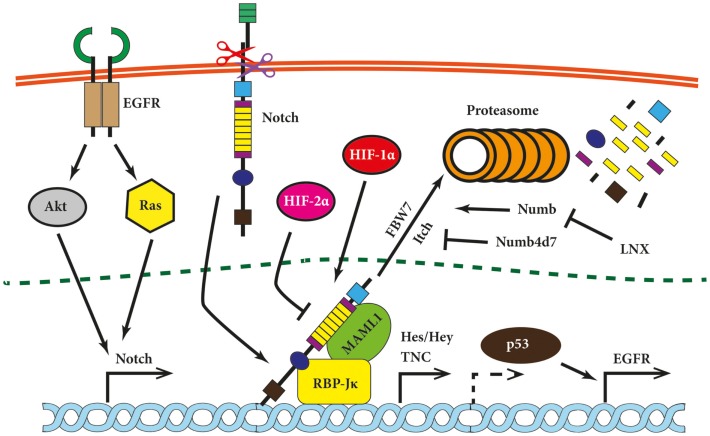
**Notch signaling modules relevant for brain tumors**. Notch signaling has been shown to be modulated on multiple levels in glioma cells and is linked upstream and downstream to other tumorigenic pathways. Its expression is induced by Ras and Akt, while Notch itself induces expression of epidermal growth factor (EGFR) *via* p53 (indirectly; hence dashed arrow) and pro-migratory glycoprotein tenascin C (TNC). Hypoxia-inducible factor 1α (HIF-1α) and HIF-2α compete for NICD binding. The Notch inhibitor HIF-2α is displaced by HIF-1α under hypoxic conditions. Further, several proteins modulate the Notch pathway at the level of NICD. Examples are Numb, which promotes Notch degradation *via* ubiquitin ligases such as FBW7 or Itch, or Numb4d7 and ligand of Notch protein X (LNX), which stimulate Notch signaling.

Notch receptors and ligands are post-translationally regulated by ubiquitylation and endocytosis. Two proteins regulating these processes have attracted attention in the cancer field, namely Neuralized1 (Neurl1) and Numb. The former is known to bind and to monoubiquinate Jagged1 in mammalian cells (90). The chromosomal fragment 10q25.1 encoding *Neurl1* has been found to be frequently lost in grade II astrocytomas and GBM. Moreover, expression of human Neurl1 was nearly absent in high-grade astrocytoma and the majority of investigated glioma cell lines in contrast to the normal brain tissue (91). Analysis of Neurl1 expression in medulloblastoma led to similar results as it was downregulated compared to normal cerebral tissue (92). Numb, another antagonist of Notch signaling, is not an ubiquitin ligase itself but rather serves as an adapter protein for the E3 ubiquitin ligase Itch (also known as AIP4) (Figure 2) (93). Overexpression of Numb led to the proteasomal degradation of NICD (94) but the degradation of Numb by ligand of Notch protein X (LNX) overcame Notch downregulation (95). Expression of LNX is reduced in gliomas of different grades (96), offering another putative mechanism for enhanced Notch signaling in brain tumors.

Notch activity is often measured by the expression levels of its direct target genes. Consequently, there are reports linking the expression of Hes/Hey transcriptional repressors to cancer prognosis. Expression of Hey-1 correlated with a twofold shorter disease-free survival compared to patients carrying Hey-1-negative tumors. In addition, Hey-1 is more frequently expressed in GBM as compared to low-grade astrocytomas while no expression was found in normal brain tissue or in neuroblastoma (97). Further, these observations have been confirmed by clinical data from 62 GBM patients, where the expression of Hey-1 correlated with a shorter overall survival. In Hey-1-negative cases, on the other hand, survival was significantly longer and accompanied by a high number of long-term survivors (98).

Non-canonical Notch ligands represent the least explored group of glioma-related markers. Dlk1 has been found to be upregulated in a subset of GBM as compared to the healthy brain. The protein has been suggested as a Notch inhibitor, which is supported by the finding that Hes-1 was downregulated in glioma cell lines stably expressing Dlk1 or treated with Dlk1-conditioned medium. Moreover, GBM cell lines transfected with this ligand exhibited an augmented proliferation (99).

## Notch in Glioma Cells

The response of brain tumor cells to the modulation of Notch signaling has been investigated by Purow and colleagues (80), who demonstrated that the siRNA-mediated knock-down of Notch1 in glioma cell lines led to an increased cell death, decreased proliferation as well as cell cycle arrest. Further, the treatment affected cell morphology and triggered the outgrowth of neurite-like extensions (80). Another group reported an upregulation of the astrocyte marker glial fibrillary acidic protein (GFAP) and a downregulation of the mesenchymal marker vimentin as well as reduced proliferation of glioma cell lines upon Notch inhibition (88). These morphologic features are indicative of differentiation, suggesting that remaining in an undifferentiated state enhances the oncogenic potential of glioma cells. These *in vitro* data suggest that Notch1 acts as an oncogene in glioma, which has been confirmed in an intracranial xenograft model, where mice injected with control U251MG cells died sooner as compared to mice that received cells in which Notch1 or Dll1 were downregulated by siRNA. Knock-down of Jagged1 did not improve survival and caused a milder inhibition of proliferation *in vitro* as compared to the Dll1 knock-down (80). Furthermore, a recent report compared the role of the Notch paralogs Notch1 and Notch2, which displayed opposing effects on the propagation of glioma cells. Equally, knocking-down Notch1 or overexpressing Notch2 suppressed cell growth and invasion in addition to enhancing apoptosis of subcutaneously engrafted U251 and A172 glioma cells (100).

Much less is known about molecules exerting a Notch-driven malignant phenotype in glioma. One report suggests that Notch expression is linked to enhanced cell migration mediated by tenascin C (TNC), whose promoter is activated by the Notch-induced transcription factor RBP-Jκ (Figure 2) (78). In brief, TNC is a matrix glycoprotein that induced proliferation and migration of neuronal precursors and its expression increases in GBM as compared to grade III astrocytomas. Moreover, patients lacking TSC in the extracellular matrix (ECM) survived significantly longer than patients with TNC-positive lesions (101).

## Notch in Stem-Like Brain Tumor-Propagating Cells

Glioblastoma, like other cancers, result from the accumulation of genetic and epigenetic mismatches (102). Until recently, it was presumed to originate solely from glial cells, i.e., astrocytes or oligodendrocytes, residing within the brain parenchyma. However, the discovery of proliferating cells in the adult brain led to a modification of this hypothesis. According to studies performed in genetically modified mouse models, gliomas may arise from NSCs or NPCs (103–105). More differentiated cells were also investigated: experiments with mosaic inactivation of *Tp53* and *Nf1* in NSCs showed that the most rapid phase of tumor growth occurs when the cells migrate out of the stem cell niche and become Olig2-positive oligodendroglial progenitor cells (106). In a different genetic model, non-stem cell progenitor cells were shown to generate astrocytomas and oligodendrogliomas upon the transgenic induction of *v-erbB* combined with *Tp53* deletion (107). Another strain utilized to investigate the tumorigenic potential of differentiated cells is *Ink4A/Arf* knock-out mice. These animals are prone to develop brain tumors as compared to wild-type mice. The *Ink4A/Arf* locus encodes two tumor suppressors: p16^INK4a^, which prevents Rb phosphorylation by binding CDK4, and p14/19^ARF^, which prevents p53 degradation *via* MDM2 inhibition. Both processes are crucial for cell cycle regulation. Astrocytes derived from these animals were shown to undergo de-differentiation and induce glioma upon the expression of the EGFRvIII oncogene or stimulation with EGF or PDGF *in vivo* (103, 108).

The term “cancer stem cell” has been coined in order to describe tumor-propagating cells (109). These cells differ from normal tissue stem cells as they lack a well-defined and conserved hierarchy (110). Further confusion is caused by referring to them as tumor-initiating cells, which is supposed to reflect that they are the origin of tumors. In our opinion, the term stem-like brain tumor-propagating cell (BTPC) is more appropriate to “cancer stem cell” or “tumor-initiating cell” in the context of glioma formation as it refers to the most solid biological feature of this cell type, namely, the propagation of glioma *in vivo*. Relying on the expression of cell markers (or the lack thereof) to name this cell type may be misleading, as the transcriptome of BTPCs is highly variable (111) and this cell type expresses all types of cellular markers (e.g., nestin, GFAP, or beta III-tubulin) in parallel (own observations). Initially, BTPCs were isolated from gliomas based on CD133 expression, propagated in serum-free medium and shown to recapitulate glioma growth *in vivo* (112). However, CD133 cannot be considered a universal BTPC marker because CD133-negative cells also give rise to tumors, and some tumors are fully devoid of CD133-positive cells (113). It should be pointed out that several groups have reported that CD133 is also expressed by endothelial cells (114–116) and might thus be better suited as a marker of enhanced angiogenesis. In support of this hypothesis, vascular CD133, but not tumor-expressed CD133, was found to correlate with glioma grade (117). Several other extracellular molecules have been proposed as BTPC markers including CD44, CD15, and integrin α6. However, they have not yet been verified by the glioma community as *bona fide* BTPCs markers (118). Furthermore, considerable attention has been paid to stem cell-specific intracellular proteins like the transcription factors Sox2, Oct-4, Bmi-1, and Id4, RNA-binding protein Musashi-1 or the intermediate filament nestin, a stem cell marker and transcriptional target of Notch (119, 120). There is evidence that the expression levels of Musashi-1 and nestin positively correlate with high glioma grade and poor survival (121).

Notch has been suggested to play an important role in the maintenance of BTPCs as it regulates the maintenance and differentiation of NSCs (122–124). First results hinted that glioma cells cultured under stem cell conditions express Notch1, Notch4, Dll1, and Dll3 (125). In another publication, nine glioma-derived cell lines were divided into two groups based on sphere-forming capacity, CD133 expression and high invasiveness. Two of the transcripts belonging to the Notch cascade were overexpressed in the tumorigenic group while their expression was not increased in any cell line from the less stem-like/invasive group (126). Overexpressing NICD in the human glioma cell line SHG-44 led to enhanced proliferation as well as higher colony- and sphere-formation potentials. Moreover, the sphere-forming cells displayed BTPC characteristics such as the expression of the NSC marker nestin and the ability to differentiate into all three neural lineages based on immunofluorescence staining for GFAP, MAP2, and GalC (89). When NICD-overexpressing BTPCs were intracranially implanted into nude mice, they formed highly vascularized tumors containing large vessels with a central lumen. The cells were, however, hardly disseminating in contrast to control cells, which infiltrated both hemispheres (127). Taken together, these results suggest that due to the activation of multiple cell responses, Notch may play different roles in BTPCs depending on the cellular and environmental context. Furthermore, intracellular modifiers of Notch signaling, such as Numb proteins, are involved in the regulation of BTPCs. Numb4 promotes Notch degradation *via* FBW7 ubiquitin ligase assembly, while its truncated form, Numb4 delta 7 (Numb4d7), generated by alternative splicing, increases Notch signaling (Figure 2). Although Numb4d7 opposes growth-inhibitory effect of Numb4, both isoforms promote expression of stem cell markers in BTPCs. Thus, it appears Numb4 can affect BTPC differentiation independent from Notch inhibition itself (128).

Lastly, microvascular proliferation is an important feature of glioblastoma, underlining the essential role of angiogenesis in brain tumor development (1, 129). Formation of blood vessels is a response to hypoxia that results in the stabilization of the hypoxia-inducible factors (HIF)-1α and -2α and the subsequent upregulation of pro-angiogenic factors like VEGF. Notch signaling has been shown to be activated by hypoxia in normal and neoplastic cells as evidenced by the increased expression of Notch1, Hes-1, Hey-1, Dll1, and Dll4 (130, 131). Notch plays a central role in maintaining NSCs undifferentiated under hypoxic conditions (132), and therefore, its impact on BPTCs has been investigated. Treatment of medulloblastoma-derived BTPCs with immobilized recombinant Dll4 under hypoxic conditions (2% oxygen) led to the expansion of CD133-positive and nestin-positive cells. Inhibition of the Notch pathway resulted in the opposite effect as tumor cells underwent neuronal differentiation when treated with the γ-secretase inhibitor (GSI) N-[N-(3,5-difluorophenacetyl)-l-alanyl]-S-phenylglycine t-butyl ester (DAPT) to inhibit Notch signaling (133). Further, the maintenance of glioma BTPCs can be hindered by the depletion of HIF-1α or inactivation of Notch signaling, partly because of the interaction between HIF-1α and NICD (134). This interaction has been confirmed by another group, which also described competition between HIF-1α and HIF-2α for NICD binding. HIF-2α inhibited Notch and was displaced by HIF-1α under hypoxic conditions (135).

## Cross-Talk of Notch with the EGFR Pathway

Overexpression and increased activity of EGFR is one of the hallmarks of primary GBM (136, 137). EGFR is a receptor tyrosine kinase that initiates multiple cellular pathways such as mitogen-activated protein kinase (MAPK) cascade or phosphoinositide 3-kinase (PI3K) pathway. Aberrant enhancement of EGFR signaling, which can be caused by overexpression, increase in gene copy number or ligand-independent mutated receptors, leads to cancer-driving processes such as augmented proliferation, angiogenesis, migration/invasion, and impaired apoptosis (138). Such activities, albeit strictly regulated, are especially crucial during ontogeny, including brain development and neurogenesis. Therefore, EGFR signaling has been investigated in the context of other neurogenic pathways such as Notch. Aguirre et al. presented evidence that EGFR and Notch have opposite effects on cells derived from subventricular zone (SVZ). Enhanced EGFR signaling caused an expansion of NPCs at the expense of self-renewing NSCs and downregulated Notch signaling via Numb (139). This antagonism ceased in the later stages of cell differentiation, as both EGFR and Notch have been shown to direct cells toward a glial fate (140, 141). Along this line, a synergy between the EGFR and Notch pathways in glioma was indicated by Purow et al. who showed that EGFR is under the transcriptional control of Notch signaling (Figure 2). Experiments involving silencing or overexpression of Notch and p53 indicate that this regulation was mediated by p53. Moreover, Notch1 and EGFR expression correlated in high-grade astrocytomas that were negative for the amplification of the EGFR gene (142).

Epidermal growth factor receptor stimulation leads to the activation of multiple signal mediators including the Ras family of small GTPases. Ras-transformed astrocytes have been shown to express higher levels of Notch1 as compared to their non-transformed counterparts and inhibition of Notch signaling reduced their aggressive phenotype. However, introduction of NICD did not lead to a transformation of the immortalized astrocytes on its own and did not enhance cell growth of Ras-transformed cells (88). The tumorigenic cooperation between Ras and Notch might occur at an earlier stage of cell development as the overexpression of NICD and K-Ras in glial progenitors induced periventricular lesions with stem-like characteristics in the SVZ (based on retention of proliferation and nestin expression). K-Ras and Notch acted in a synergistic manner in this model as the individual overexpression of each did not cause a comparable phenotype (119). Further evidence showed that both Ras and Akt induce Notch1 expression in a mouse glioma model (143).

## Notch-Based Brain Tumor Therapies in Clinical Trials

### γ-Secretase inhibitors

Brain tumor-propagating cells have been shown to exhibit a higher resistance to chemo- and radiotherapy than bulk tumor cells (144, 145). However, they seem equally sensitive to treatments targeting stem cell pathways such as Notch (146, 147). Cleavage of Notch receptors and the formation of the NICD fragments is inhibited by GSIs in order to shut off Notch signaling (Figure 1). Several of them have been successfully applied in animals. The first GSI tested in a brain tumor model was DAPT. Treatment of mice harboring D283 medulloblastoma xenografts with DAPT resulted in decreased proliferation and increased apoptosis of tumor cells (148). The promising results of DAPT treatments prompted the development and testing of other inhibitors such as GSI-18 (149). This drug has been tested in two different subcutaneous brain tumor models. In the first setting, pretreatment of DAOY medulloblastoma cells hindered the growth of subcutaneous xenografts (147). The same group applied tumorspheres isolated from primary GBMs for subcutaneous implantation studies. Spheres pre-treated with GSI-18 did not form tumors anymore. These observations were confirmed in intracranial implantation studies in a relatively small cohort of animals. Mice treated with GSI-18 survived significantly longer post-engraftment as compared to the control group (146).

Chemotherapy is often based on a combination of drugs and thus GSIs have been tested together with already established anti-tumor agents. *Ex vivo* treatment with TMZ and DAPT lowered the tumorigenicity of U87NS and U373NS cells in subcutaneous mouse xenograft models. Moreover, administration of GSI (LY411, 575) and TMZ *in vivo* blocked tumor progression in four out of eight mice carrying U87NS xenografts (150). Administration of TMZ plus RO4929097 (Roche) was partially effective in mice intracranially injected with Hs683 cells. The authors identified the ECM protein EFEMP1 as a mediator of TMZ resistance and a target of the GSI. Further, the analysis of publicly available glioblastoma databases revealed that EFEMP1 expression correlated with the resistance to TMZ treatment and such could serve as a marker for the application of GSIs (151). GSIs (DAPT and L685, 458) were also shown to dramatically increase cell death of irradiated glioma BTPCs *in vitro*. This phenotype was reversed by the overexpression of NICD of Notch1 or Notch2, confirming that Notch is essentially involved in the survival of glioma BTPCs post irradiation. Furthermore, siRNA-mediated knock-down of Notch1 or Notch2 impaired tumor formation in xenografts but unfortunately, GSIs were not tested in this setting (152).

Several GSIs have been tested in clinical studies in various tumor types (Table 1). RO4929097 has been shown to extend the median survival rate in an intracranial mouse glioma model (153). This GSI has completed several phase I studies: applied alone (154), in combination with the VEGF tyrosine kinase inhibitor ceridanib (155) or in combination with gemcitabine (156). Additionally, a phase Ib trial tested the combination of RO4929097 and the mTOR inhibitor temsirolimus (157). All four studies showed that the drug is tolerable, and few patients showed a partial response. Although patients bearing a broad range of solid tumors were recruited, the drug was not tested on brain tumor patients. Currently, there are three ongoing trials directed against malignant glioma: a phase I study that combines the GSI RO4929097 with TMZ and radiation therapy (NCT01119599), a phase I/II study in which the drug is tested in combination with bevacizumab, a monoclonal anti-VEGF antibody (NCT01189240) and applied alone in a phase II trial (NCT01122901). The outcome of these treatments still awaits publication but the results of two different phase II cancer studies have been published. Unfortunately, none of 33 treated patients with colorectal metastatic cancer showed an objective radiographic response and the median progression-free survival was only 1.8 months in total (158). The outcome of a study on patients previously treated for metastatic pancreatic carcinoma did not meet its goals either. Consequently, the development of RO4929097 was discontinued by the sponsor (159).

**Table 1:** **Clinical trials involving GSIs and relevant for brain tumor therapy**.

Compound	Co-treatment	Phase	Status	Tumor type	Identifier
BMS906024	Paclitaxel, 5-fluorouracil (5FU), carboplatin, leucovorin, irinotecan	I	Recruiting	Advanced or metastatic solid tumors	NCT01653470
BMS906024		I	Recruiting	Advanced or metastatic solid tumors	NCT01292655
LY900009		I	Completed	Advanced cancer	NCT01158404
**MK-0752**		**I**	**Completed**	**Advanced solid tumors**	**NCT00106145**
MK-0752	Ridaforolimus	I	Ongoing	Advanced cancer	NCT01295632
**MK-0752**		**I**	**Terminated**	**Pediatric CNS cancer**	**NCT00572182**
MK-0752	Dalotuzumab	I	Terminated	Advanced cancer	NCT01243762
PF-03084014		I	Ongoing	Advanced cancer	NCT00878189
**RO4929097**	**Temozolomide, radiation**	**I**	**Ongoing**	**Malignant glioma**	**NCT01119599**
**RO4929097**		**I**	**Terminated**	**Recurrent invasive gliomas**	**NCT01269411**
RO4929097	Capecitabine	I	Ongoing	Refractory solid tumors	NCT01158274
RO4929097	Gemcitabine hydrochloride	I	Completed	Refractory solid tumors	NCT01145456
**RO4929097**	**Cediranib maleate**	**I**	**Ongoing**	**Advanced solid tumors**	**NCT01131234**
RO4929097		I	Completed	Metastatic or unresectable solid malignancies	NCT01096355
RO4929097	Temsirolimus	I	Completed	Metastatic or unresectable solid malignancies	NCT01198184
**RO4929097**	**Dexamethasone**	**I**	**Withdrawn**	**Pediatric solid tumors, CNS tumors**	**NCT01236586**
RO4929097	Ketoconazole, rifampin, midazolam hydrochloride, omeprazole, tolbutamide, dextromethorphan hydrobromide	I	Ongoing	Advanced solid tumors	NCT01218620
RO4929097		I	Completed	Advanced solid tumors	NCT00532090
**RO4929097**	**Stereotactic radiosurgery, whole-brain radiation therapy**	**I/II**	**Terminated**	**Breast cancer-derived brain metastases**	**NCT01217411**
**RO4929097**	**Bevacizumab**	**I/II**	**Ongoing**	**Progressive or recurrent malignant glioma**	**NCT01189240**
**RO4929097**		**I/II**	**Terminated**	**Solid tumors, CNS tumors**	**NCT01088763**
**RO4929097**		**II**	**Ongoing**	**Recurrent or progressive glioblastoma**	**NCT01122901**

***Bold**: explicitly, brain tumors have been mentioned in these studies*.

MK-0752 is a GSI designed by Merck and has been tested in several phase I studies. It was applied to a cohort of 103 patients with solid tumors. Weekly dosing was well tolerated and led to the modulation of the expression of nine Notch-related genes referred to as Notch gene signature. This study generated promising results as one patient with anaplastic astrocytoma had a complete remission lasting for more than 1 year in addition to a stable disease progression lasting for more than 4 months in 12 patients. All 12 patients were affected by brain malignancies and 10 of them were diagnosed with glioma (24% of all glioma patients in the study) (160). Further, MK-0752 was well tolerated by children with recurrent central nervous system malignancies (161). GBM patients were also included in an ongoing phase I trial involving the combination of MRK-0752 and ridaforolimus, a small-molecule mTOR inhibitor (NCT01295632). The last clinically tested GSI is PF-03084014 from Pfizer, currently in phase I for a wide range of solid and leukemic tumors (NCT00878189) and in the recruiting phase for several other trials but no results have been published so far.

It should be noted that administering GSIs raises several concerns. First of all, γ-secretases are involved in the cleavage of a multitude of proteins (162, 163). Furthermore, animal studies and phase I clinical trials have shown that systemic drug distribution leads to gastrointestinal toxicity, caused by the accumulation of secretory goblet cells in the intestine and due to the inactivation of Notch signaling (164–166). However, animal experiments indicate that these side-effects may be ameliorated by the application of glucocorticoids (167).

### Blocking Antibodies

Several antibodies blocking Notch activity have been developed for the treatment of brain tumors due to their higher specificity as compared to GSI inhibitors. Furthermore, they can be used to block individual Notch receptors and ligands. Treatments using specific antibodies offer the advantage of fewer side-effects as compared to GSIs. Accordingly, agents neutralizing Notch1, Notch2, or Dll4 did not affect intestinal goblet cell differentiation in mice (168, 169).

Blocking antibodies targeting Notch receptors can be divided into two groups (Figure 1). Members of the first group are directed against the NRR block the receptor conformation that allows ADAM cleavage. Such antibodies have been raised against Notch1 (NRR1), Notch2 (NRR2), and Notch3 (NRR3) and have been tested in pre-clinical as well as *in vitro* studies (169–171). The other approach is to block Notch receptor–ligand interactions by hindering EGF repeats required for binding (170). The antibodies of this group are also effective against receptors carrying NRR mutations that destabilize the auto-inhibited receptor conformation and cause constitutive ligand-independent Notch signaling. Based on the success of pre-clinical studies, a humanized antibody targeting Notch1 (OMP-52M51) and Notch2/Notch3 (OMP-59R5; both OncoMed Pharmaceuticals) have entered a dose escalation phase I study in patients with solid tumors (NCT01778439, NCT01277146) followed by phase Ib/II trials in pancreatic and lung cancer patients in case of OMP-59R5 (Table 2).

**Table 2:** **Clinical trials involving Notch signaling-specific antibodies and relevant for brain tumor therapy**.

Compound	Target	Phase	Status	Tumor type	Identifier
MEDI0639	Dll4	I	Recruiting	Advanced solid tumors	NCT01577745
OMP-21M18	Dll4	I	Completed	Solid tumors	NCT00744562
REGN421	Dll4	I	Completed	Advanced solid tumors	NCT00871559
OMP-52M51	Notch1	I	Recruiting	Solid tumors	NCT01778439
OMP-59R5	Notch2, Notch3	I	Ongoing	Solid tumors	NCT01277146

Out of all Notch ligands, the blocking of Dll4 has been most thoroughly investigated. This protein plays an important role in angiogenesis and blocking the Dll4-Notch interaction (by overexpression of a soluble Dll4-Fc decoy peptide) enhanced vascular density and angiogenic sprouting in tumors derived from the rat glioma line C6. Surprisingly, this vasculature was rendered non-functional as subcutaneous xenografts of Dll4-Fc-treated cells grew smaller in nude mice. A similar response in C6 tumors was induced upon systemic delivery of Dll4-Fc using an adenoviral overexpression system. Further, the authors developed the first pharmacological model for anti-Dll4 treatment, i.e., using defined amounts of systematically delivered recombinant agent. Administration of recombinant Dll4-Fc or anti-Dll4 polyclonal antibody in an HT1080-RM (generated from a bevacizumab-resistant human fibrosarcoma) tumor model caused an increase in vessel density and smaller tumors volumes (172). These results were recapitulated by Li et al. in the human glioma cell line U87 subcutaneously implanted in nude mice. The authors showed that the overexpression of dominant negative soluble Dll4ECD-Fc enhanced the number of blood vessel and reduced tumor growth *in vivo*. Accordingly, the ectopic expression of Dll4 caused the opposite response. Moreover, examination of 20 surgical GBM specimens yielded an upregulation of Dll4 both in GBM tumor cells as well as endothelial cells (173). The same tumor model was applied to identify Dll4 as a mediator of tumor resistance to anti-VEGF therapy. According to data presented, the Dll4-mediated formation of larger vessels insensitive to anti-VEGF treatment was responsible for the resistance observed (174). Anti-Dll4 antibodies have already entered clinical trials (Table 2). The pharmacokinetics of three of them, MEDI0639 (175) (Astrazeneca, NCT01577745), OMP-21M18 (OncoMed Pharmaceuticals, NCT00744562), and REGN421 (Regeneron Pharmaceuticals, NCT00871559) have been tested in solid tumors in phase I studies but the results have not yet been published. However, it should be noted that side-effects of manipulating Dll4-induced Notch signaling have been reported in animal studies. One study showed that the treatment with anti-Dll4 antibody led to pathological changes in rat livers and the formation of vascular neoplasms, resembling hemangioblastomas (176). Another group reported chimeric Dll4 to affect hematopoiesis by inhibition of megakaryocyte differentiation (177).

### Decoys

Soluble extracellular domains of Notch receptors and ligands offer an alternative to blocking antibodies (Figure 1). A Notch1 decoy has been shown to act as a pan-ligand antagonist, which can be applied to block Notch signaling in endothelial cells. Even though the decoy did not affect mouse breast cancer or human neuroblastoma cells *in vitro*, its overexpression in engrafted cells decreased tumor viability and disrupted vessel formation *in vivo* (178, 179). Soluble forms of Dll1 (180), Dll4 (172, 181), and Jagged1 (182) have also been used to block Notch signaling. Endogenous, soluble Notch antagonists like Dll1ECD (183) are either the product of metalloproteinases or are *bona fide* secreted proteins such as EGFL7, a novel non-canonical soluble Notch ligand with established neurovascular implications. The protein has been shown to act as an antagonist of Jagged1 and inhibited Notch activity in NSCs (34) and primary endothelial cells (184), thus offering a promising tool for the manipulation of glioma formation. In a genetically engineered mouse model of lung cancer, blocking ECM-associated EGFL7 with the antibody m18F7 (Genentech) enhanced progression-free and overall survival induced by anti-VEGF therapy (185). Moreover, silencing of EGFL7 by siRNA *in vitro* has been shown to inhibit the adherence of endothelial cells to a collagen-coated semipermeable membrane in a co-culture system with U251 glioma cells (186).

### Other Agents

Certain natural compounds have been shown to affect the Notch signaling pathway and, even though their mode of action is not entirely known, they have been tested for therapeutic purposes. Curcumin, for example, has been shown to hinder proliferation and invasion of osteosarcoma cells *in vitro* by inhibiting Notch signaling (187). Several publications report enhanced apoptosis in glioma cell lines and decreased tumor growth *in vivo*. In most of these reports, the effect on Notch pathway activation has either not been mentioned at all (188) or has not been observed (189). Regardless of the mode of action a phase I study that measured curcumin bioavailability in glioblastoma patients has been completed (NCT01712542). Further natural compounds such as resveratrol and inducing apoptosis in glioma cells have been discussed elsewhere (190). The last group of compounds to be discussed here includes peptides affecting intracellular Notch signaling and small RNA molecules. A stapled peptide that mimics MAML1 binding to NICD–RBPJ has been shown to disrupt the ternary transcription complex and to pass through the cell membrane (191). Although RNA interference is still mostly a tool for basic research, it should be noted that siRNA targeting mutant KRAS in pancreatic cancer passed phase I (192) and entered phase II of clinical trials (NCT01676259).

## Concluding Remarks

The improved understanding of genetics and molecular biology has increased the number of novel anti-glioblastoma therapies in clinical trials. The Notch signaling pathway is increasingly recognized as a central player in brain tumor formation beyond targeting the “usual suspects” such as EGFR and PI3K. One reason is that the oncogenicity of Notch can be connected to the maintenance of an undifferentiated cell state of brain tumor cells. Altering Notch signaling might lead to the differentiation of cancer cells, while targets such as EGFR or PI3K target cell proliferation only. In the former case, one might get rid of the tumor, while it just grows more slowly in the latter. Consequently, a combination of both strategies could be fruitful. The earliest efforts to modulate the Notch pathway focused on GSIs. Despite concerns regarding side-effects, several compounds have completed phase I of clinical studies and are awaiting further testing. The means to circumvent drawbacks of systemic administration of GSIs are still emerging. One possibility is the targeted delivery of a drug, e.g., via silica nanoparticles (193). Advances in recombinant protein technologies offer further possibilities to modulate Notch signaling, and consequently, reagents such as Notch blocking antibodies are entering clinical trials, while decoys have already been tested in animals. Their specificity might prove advantageous over small molecules and it should be noted that techniques previously considered inapplicable, such as siRNA technology, are now being tested in patients as well. Moreover, blocking Notch transcription factors with cell-permeable peptides offers a promising alternative for brain tumor therapy. The future of cancer treatment lies in personalized regimens and the combination of treatments. The means for molecular diagnostics are rapidly advancing and Notch-targeting drugs are the focus of various clinical trials; therefore, the future is bright for Notch-based brain tumor therapies.

## Conflict of Interest Statement

The authors declare that the research was conducted in the absence of any commercial or financial relationships that could be construed as a potential conflict of interest.
